# Canary: an automated tool for the conversion of MaCH imputed dosage files to PLINK files

**DOI:** 10.1186/s12859-022-04822-8

**Published:** 2022-07-27

**Authors:** Adam N. Bennett, Jethro Rainford, Xiaotai Huang, Qian He, Kei Hang Katie Chan

**Affiliations:** 1 Jockey Club College of Veterinary Medicine and Life Sciences, City University of Hong Kong, Hong Kong SAR, China; 2 https://github.com/jethror1; 3 School of Computer Science and Technology, Xidian University, Xi’an, 710071 Shaanxi China; 4 Department of Biomedical Sciences, City University of Hong Kong, Hong Kong SAR, China; 5 Department of Electrical Engineering, City University of Hong Kong, Hong Kong SAR, China; 6 Department of Epidemiology, Centre for Global Cardiometabolic Health, Brown University, Providence, RI USA

**Keywords:** MaCH, Imputed data, GWAS, Dosage file, PLINK

## Abstract

**Background:**

Previous studies have demonstrated the value of re-analysing publicly available genetics data with recent analytical approaches. Publicly available datasets, such as the Women’s Health Initiative (WHI) offered by the database of genotypes and phenotypes (dbGaP), provide a wealthy resource for researchers to perform multiple analyses, including Genome-Wide Association Studies. Often, the genetic information of individuals in these datasets are stored in imputed dosage files output by MaCH; mldose and mlinfo files. In order for researchers to perform GWAS studies with this data, they must first be converted to a file format compatible with their tool of choice e.g., PLINK. Currently, there is no published tool which easily converts the datasets provided in MACH dosage files into PLINK-ready files.

**Results:**

Herein, we present *Canary* a singularity-based tool which converts MaCH dosage files into PLINK-compatible files with a single line of user input at the command line. Further, we provide a detailed tutorial on preparation of phenotype files. Moreover, *Canary* comes with preinstalled software often used during GWAS studies, to further increase the ease-of-use of HPC systems for researchers.

**Conclusions:**

Until now, conversion of imputed data in the form of MaCH mldose and mlinfo files needed to be completed manually. *Canary* uses singularity container technology to allow users to automatically convert these MaCH files into PLINK compatible files. Additionally, *Canary* provides researchers with a platform to conduct GWAS analysis more easily as it contains essential software needed for conducting GWAS studies, such as PLINK and Bioconductor. We hope that this tool will greatly increase the ease at which researchers can perform GWAS with imputed data, particularly on HPC environments.

## Background

Publicly available, large cohort studies have been instrumental in the rise of Genome-Wide Association Studies (GWAS) as they provide a wealth of data that would otherwise be extremely difficult to obtain. Specifically, these studies collect the genetic information of participants as well as a vast amount of phenotypic data including disease outcomes. Hence, researchers can perform various statistical analyses, including GWAS, on these data in the hopes that they can identify genetic variation which contributes to disease risk [1–3]. Re-analysing publicly available data with various genetic resources and analytical methodologies can yield novel insights into genetic pathophysiology and genetics of complex diseases [4]. These data are made available through online databases such as the database of genotypes and phenotypes (dbGaP) provided by the National Institute of Health (NIH) [5] and the UK Biobank (UKB). Due to the high cost of whole-genome sequencing (WGS), single-nucleotide polymorphism (SNP) genotyping arrays are most often used in large cohort studies. As these arrays only capture between 300,000 and 1 million single nucleotide variants (SNVs), and not the whole genome, imputation is used to increase the genomic coverage [6]. To do this, linkage disequilibrium is utilised to estimate untested SNPs using a reference genome of an appropriate population [7]. Currently, there are several imputation tools available, including MaCH [8], minimac, Impute2 [9], and BEAGLE [10]. MaCH and minimac are closely related methods and are amongst the most popular tools used. When calculating dosage of SNPs, both MaCH and minimac output the data into mldose and mlinfo files which store the dosage of each SNP per individual and information regarding the SNPs, respectively. Imputation methods can be performed locally but it is not always feasible due to the large size of the files involved. Therefore, researchers are often required to use a High-Performance Computing (HPC) system. However, using an HPC system has its own limitations such as not being able to access the internet during jobs and are also not very beginner friendly as they require the user to have competency with Linux environments. In attempts to overcome these barriers, researchers have developed online tools, e.g., the Michigan Imputation Server, which allow researchers to use a Graphical User Interface (GUI) to perform the imputation online with relative ease. However, due to data privacy issues, researchers may be unable to upload their data to off-site facilities like the Michigan Imputation Server and therefore must either perform the imputation locally or on their own HPC.

**Fig. 1:**
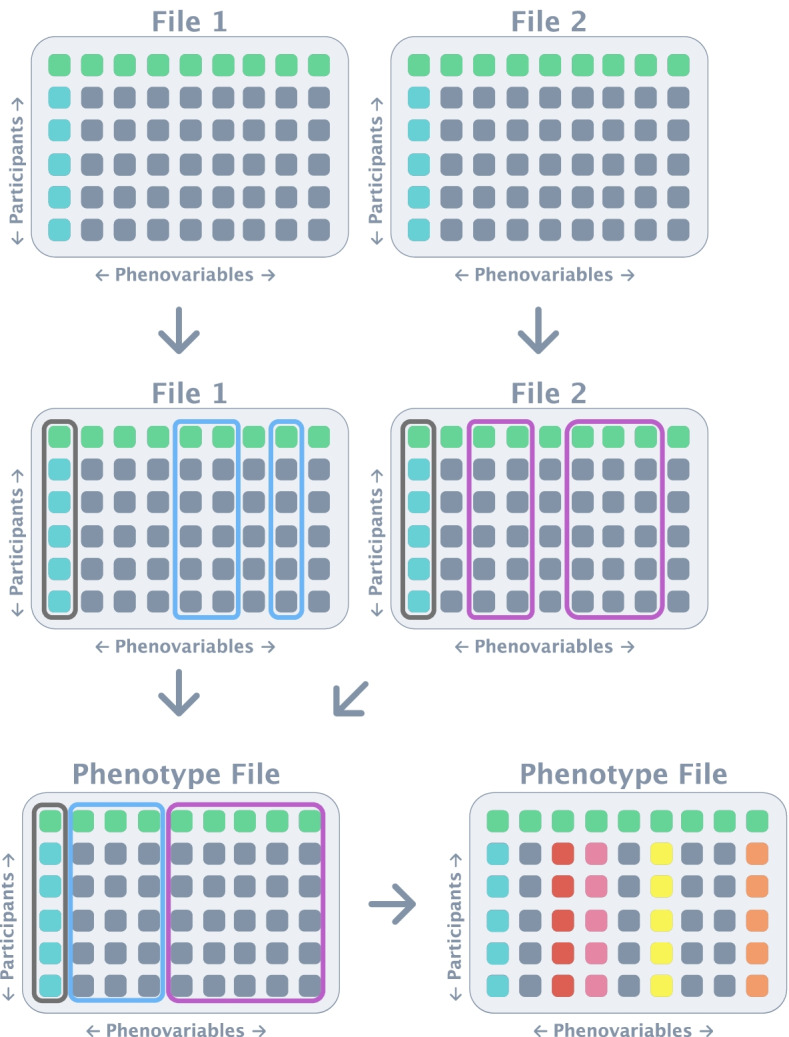
Illustration of phenotype files available in large cohort datasets. There are multiple files that contain the phenotypic information but for the sake of simplicity here we only show two. The green squares represent the header of the file which contains the column names, and the turquoise-coloured squares represent each individual in the study (i.e. the participant ID). In a study, we may wish to use data stored in the columns outlined in blue in File 1 and the columns outlined in purple in File 2. The researchers would combine the files into a new file using the participant ID. Once the phenotype file is created, the data often needs to be pre-processed before use. For example, in the WHI dataset, occurrence of cancer is recorded as number of days into the trial which cancer was discovered, however most studies will need this information in the format of age diagnosed

Many of the aforementioned large cohort studies, including the Women’s Health Initiative (WHI), provide the data in MaCH/minimac imputed dosage files. However, in order to use these dosage files in GWAS analyses, they must first be converted into file types compatible with the tool that the researcher intends on performing the analysis with. Currently, multiple tools are available for performing GWAS, including GWASTools [11], SNPTest [12], and PLINK [13] with the latter being one of the most popular tools. Although highly complex tools that can perform multiple steps automatically, including imputation and GWAS, have been published previously they do not accommodate pre-imputed data provided by dbGaP [14]. It is worth noting, however, that there have been tools developed which are able to convert MaCH mldose and mlinfo files, into PLINK format, most notably dose2plink, developed by Sarah Medland using the Perl language [15]. While this tool does allow users to convert MaCH dosage files into PLINK-compatible files, it outputs each chromosome file into multiple smaller files, which only contain small segments of each chromosome. Because of this, users must manually combine each of these files to form each chromosome. Dose2plink was subsequently rewritten in the C programming language by Christopher Chang (dose2plink.c) and this limitation was removed, outputting only a single file per chromosome [16]. Additionally, as dose2plink.c is written in C, a compiled language, it is substantially faster than the original dose2plink. However, this also means that the tool must be downloaded and compiled by each user before they can use it, which can present some difficulties for users. In recent years, imputation on new datasets has been performed using minimac rather than MaCH, and DosageConverter, developed by Sayantan Das, has been used to convert these file sets into PLINK and other file types [17]. Nevertheless, DosageConverter is not able to convert older imputed datasets, in mldose and mlinfo format, into PLINK compatible files. To our knowledge, no currently published tool provides automatic conversion of MaCH mldose and mlinfo files directly to PLINK input files [14]. Herein, we present *Canary* a tool which automatically converts mldose and mlinfo files from one or more projects into to PLINK1 [13] and PLINK2 [18] input files. We include the ability to convert to both PLINK1 and PLINK2, as of the time of writing, PLINK2 is currently in the alpha stage of development. Because of this, PLINK2 lacks several key functions that users may still need to use PLINK1. However, it is advised that PLINK2 is used as much as possible as it preserves the original dosage information in the dataset to approximately 4 decimal places, whilst PLINK1 does not. Though, it is worth noting that as PLINK1 uses 2 decimal places it can allow for a clearer comparison. Furthermore, PLINK1 is not always the most suitable choice when filling a gap currently left by PLINK2 functionality. For example, it is better to merge datasets with BCFTools, which is also included in Canary, as it can preserve the dosage information. We hope that this tool that we developed will allow users to more easily analyse existing imputed datasets [19, 20]. Of note, as the datasets compromise of both genotype and phenotype data, both must be prepared before performing GWAS. However, preparing the phenotype files (Fig. 1) automatically does not fall within the scope of this tool. Instead, we provide a step-by-step tutorial for preparing these files using Python3. *Canary* is a singularity container which comes with many preinstalled software, including dose2plink.c, which allows users to use directly on any system, with singularity installed, without any further installation themselves.

## Implementation

Herein, we demonstrate using *Canary* with the Women’s Health Initiative (WHI) data as an example dataset [21]. The WHI dataset is comprised of two consent groups, each with their own terms of use (non-profit related research only etc.). Additionally, within the WHI there are several sub-studies, including SHARE, GARNET and WHIMS. Regarding the genomic data, for each sub-study, the SNP dosage information per individual is stored in a single.dose file per chromosome whereas information about the SNPs is stored in a single.info file per chromosome. The phenotype data for the study consists of multiple tab-delineated files that contain information about the participants that were collected during the study, such as age, ethnicity, disease occurrence (e.g., cancer or diabetes) and so on. In-order-to use these files in a GWAS, the phenotypes of relevance to the study must first be collated.

## Phenotype file generation

The automatic combination and pre-processing of phenotypic data is extremely complex and is therefore not within the scope of this tool. However, we provide a python script, “Tutorial-for-Phenotype-File.py” which acts as a step-by-step tutorial for the user to generate their own python script for collating and pre-processing the data they wish to use. In brief, the required files are loaded into the python environment and pandas is used to extract the columns based on the column name. These extracted columns are combined into a new data frame using the ‘Sample_ID’ column, as each row represents an individual within the study. As occurrence of disease is recorded as number of months into the study that the participant was diagnosed with the disease, we also demonstrate how to transform this data into the age at which the patient was diagnosed. Of note, this script also demonstrates how to generate a covariate file with the phenotype information and the PCA information provided in the dataset.

## Genotype file conversion overview

**Fig. 2:**
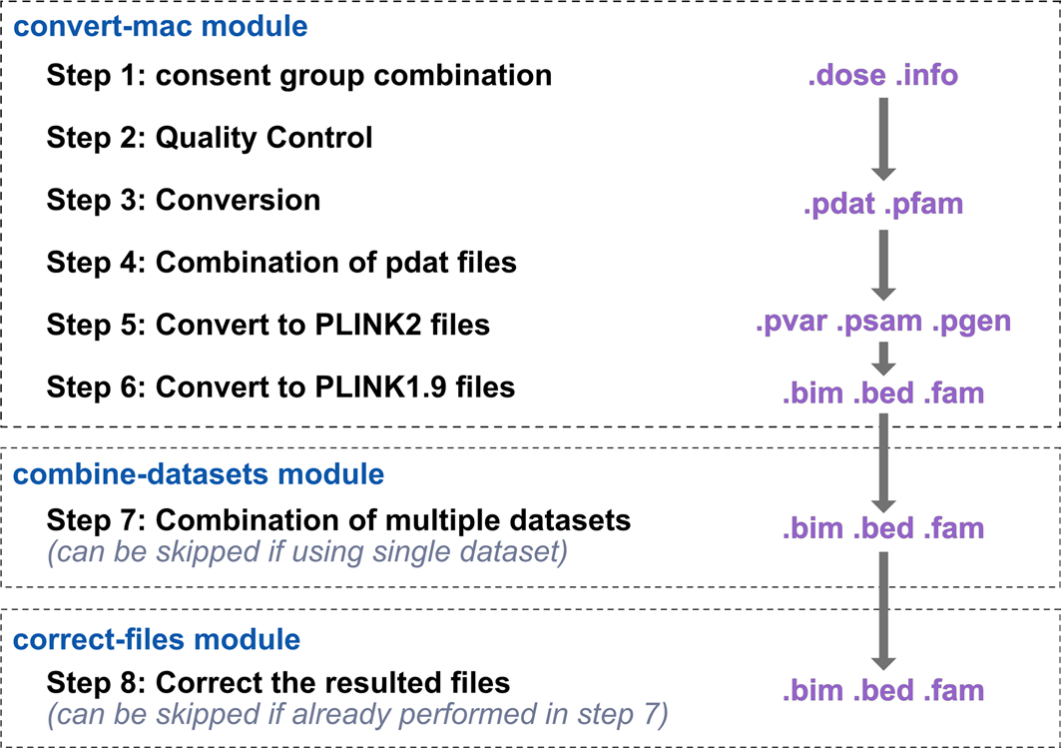
Overview of genotype data modules. The convert-mac module (top) converts the MaCH/minimac dosage files and converts them into plink compatible files; bim, bed and fam files. The combine-datasets module (middle) allows the user to combine multiple datasets together, however, this data must be harmonised before using this module. The correct-files module is the final step in the process, which updates the plink files to contain the correct information as some information is lost during the conversion process

*Canary* is capable of automatically converting the imputed dosage files into PLINK compatible files. To achieve this, *Canary* has 3 modules which complement each other. An overview of the 3 modules of Canary can be seen in Fig. 2.

**Fig. 3:**
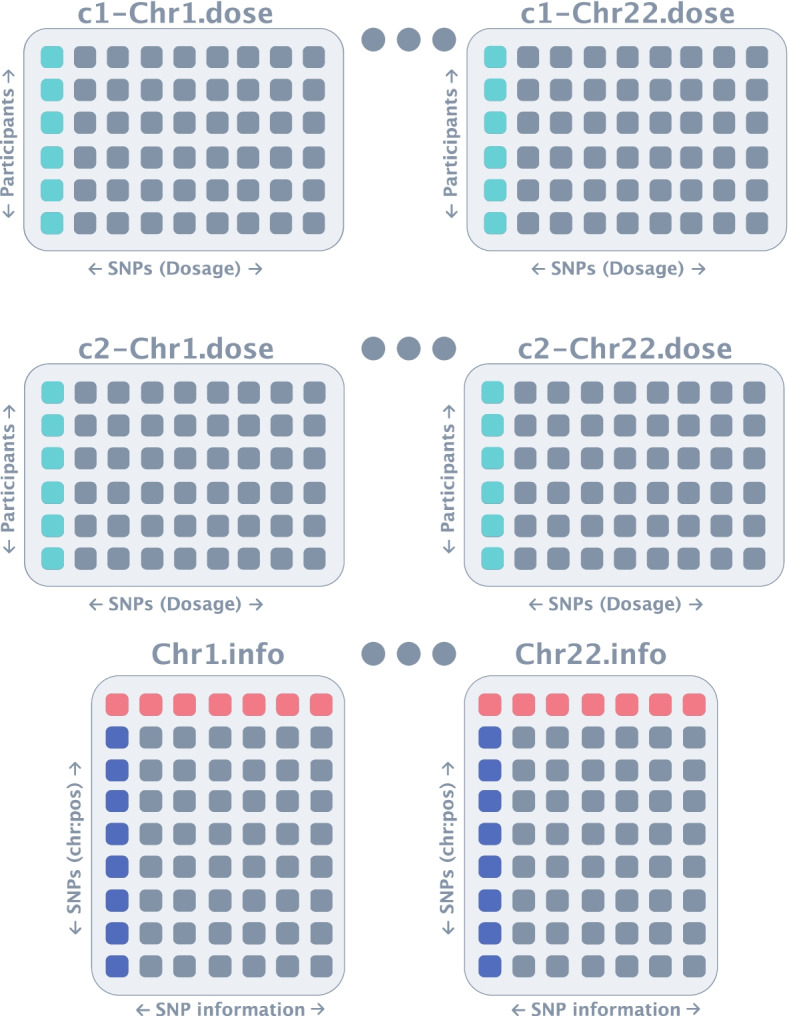
Genotype files provided by dgGaP for WHI. The.dose files, the first column (turquoise) represents each individual in the dataset and each subsequent column is the dosage of a given SNP. The.info file contains detailed information regarding the SNPs in the study, where each row is a SNP denoted by its chromosome and position) (dark blue)

## Convert-mac module

**Fig. 4:**
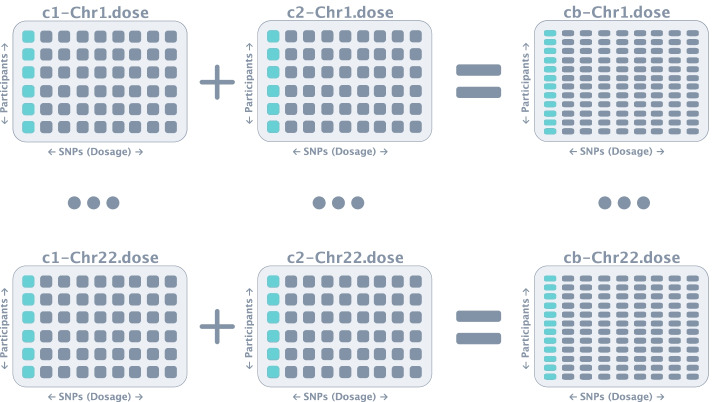
Consent group combination. In large cohort studies, there are typically multiple consent groups which agree to their data being used for different purposes. University researchers will most often have access to both consent groups and thus the consent groups need to be combined first. In this step, the chromosomes in each consent group are combined i.e. consent group 1 chromosome 1 with consent group 2 chromosome 1 etc. In this example there are only 2 consent groups, but *Canary* is capable of combining as many as required

**Fig. 5:**
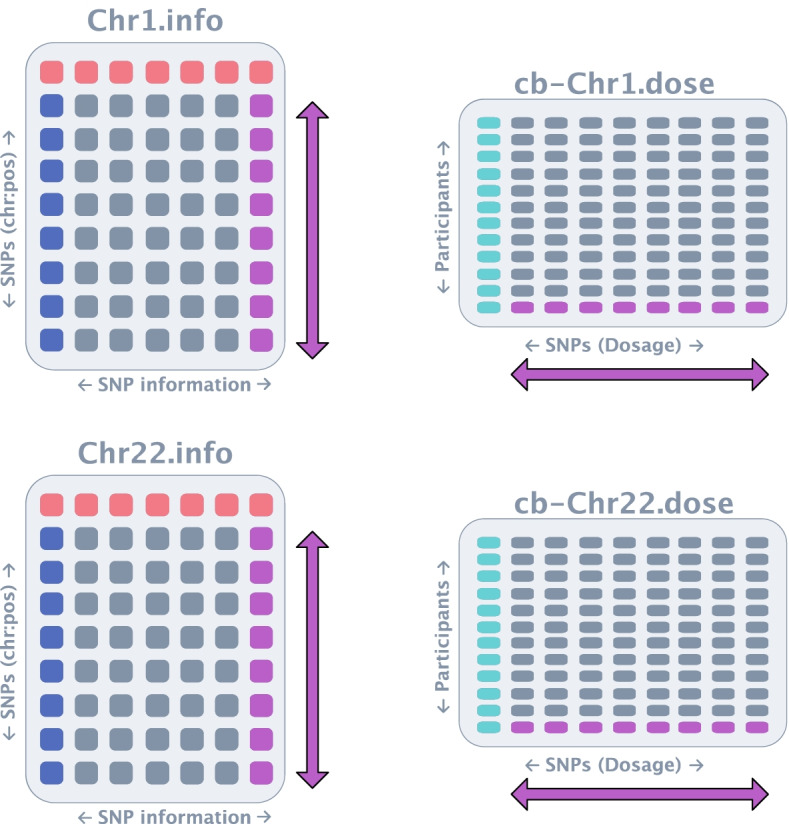
Quality Control for consent group combination. To ensure the combination of the consent groups occurred successfully, a quality control step is required. To do this, *Canary* outputs the number of lines in the info file and compares it to the number of columns in the dose file: there should be n + 1 number of columns in the dose file in comparison to the info file as the first 2 columns in the info file do not contain dosage information whereas the info file only has 1 row, the header, which does not contain SNP information. Red squares in the info file denotes the file header

**Fig. 6:**
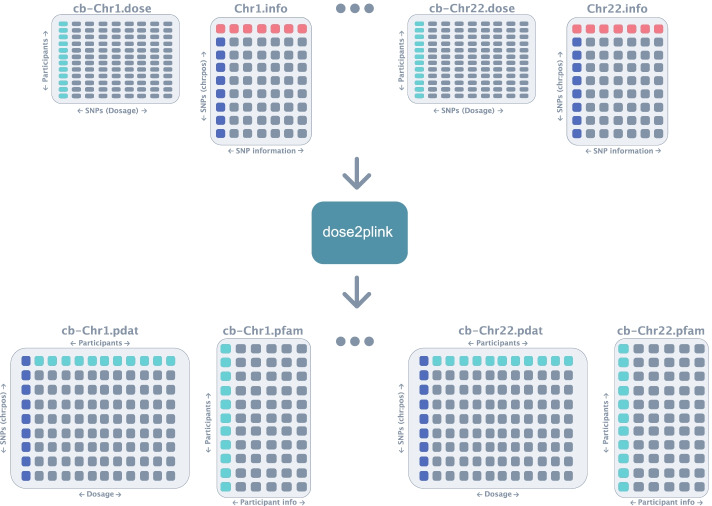
Conversion of files. In this step, *Canary* uses dose2plink.c, previously developed by other research, to convert the dose and info files into pdat and pfam files

**Fig. 7:**
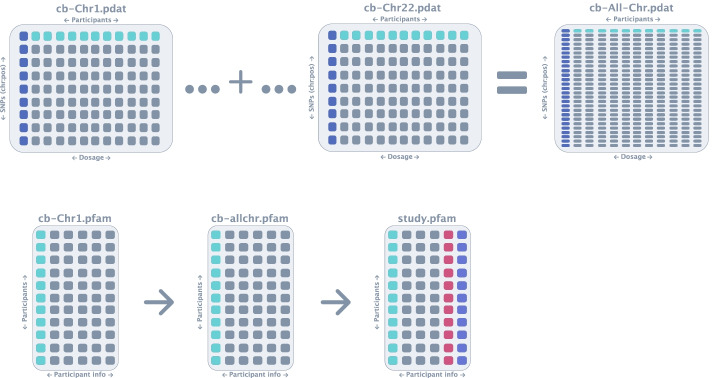
Combination of pdat files. In this step the pdat file for each chromosome into a single pdat file. As all pfam files are identical, the pfam file for chromosome 1 is copied and renamed

**Fig. 8:**
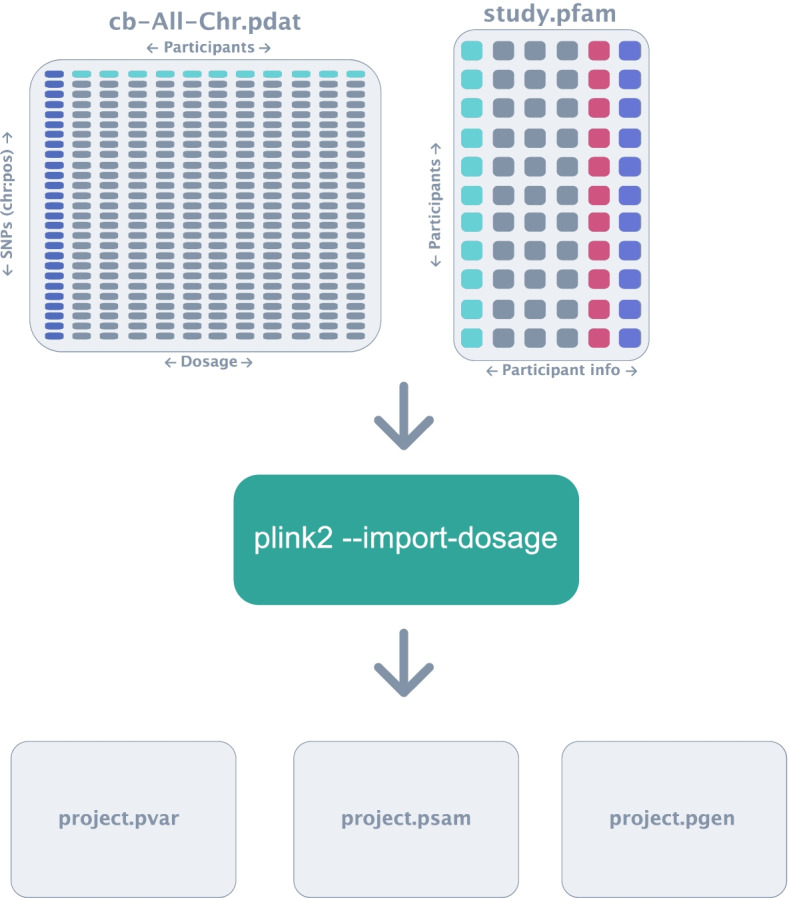
Importing pdat and pfam files into PLINK2 compatible files. During this step, *Canary* uses the PLINK2 function import dosage to convert the pdat and pfam files into PLINK2 files

The convert-mac module of *Canary* performs the steps demonstrated in Figs. 3, 4, 5, 6, 7, 8 automatically with a single command line argument which specifies: the input file directories, desired project name, and output directory. Importantly, this module of *Canary* deals with a single sub-study at a time. In the initial step, *Canary* combines the consent groups by combining each of chromosome dose files i.e., consent group 1 chromosome 1 with consent group 2 with chromosome 1 (Fig. 4). It is worth noting that, whilst this specific dataset only requires the combination of 2 consent groups, *Canary* is capable of combining as many as required by the user. A QC check is then performed to make sure that the combination was without error, and the output is saved to a file in the output directory for the user (Fig. 5).

Once the consent groups have been combined, *Canary* uses dose2plink.c previously developed by other research to convert the dose and info files into pdat and pfam files in PLINK format [16] (Fig. 6). In order to build dose2plink within the *Canary* container, dose2plink was modified; further details can be found on the GitHub repo for the modified tool [22]. dose2plink.c was chosen over the original dose2plink due to the higher efficiency of the C language in comparison to Perl, and due to the fact that dose2plink.c creates a single output per chromosome instead of producing small segments of each chromosome which then need to be combined.

**Fig. 9:**
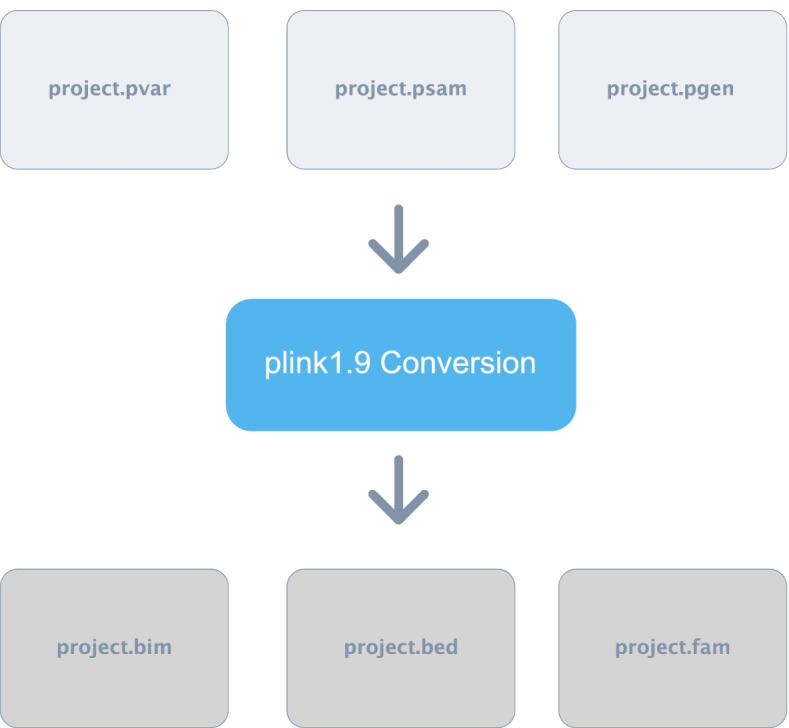
Conversion of files into PLINK1.9 compatible files. The files are converted into PLINK1file types as this version of PLINK is a lot more stable and currently provides a lot more features

The next step performed by *Canary* is to combine all of the pdat files into a single file so that there is a single pdat file for the whole dataset (Fig. 7). Additionally, the information describing individuals is stored in the pfam file, and since each of the chromosomes contain the same participants, *Canary* makes a duplicate of the pfam file for the first chromosome and renames it to reflect the name of the study. The resulting pdat and pfam files are then passed through the import dosage function of PLINK2 to produce pvar, psam and pgen files which can be used directly with PLINK2 for GWAS analysis (Fig. 8). However, due to the fact that PLINK2 is currently in alpha stage and full functionality is not yet fully available, *Canary* also uses PLINK2 to output the files in the format compatible with PLINK1.9 i.e., bim, bed and fam files (Fig. 9).

## Correct-files module

Importantly, during the conversion process the resulting PLINK files do not retain the correct chromosome and position information. Hence, *Canary* also contains a module which uses the PLINK files as a template to produce an updated file set which fixes these errors and therefore are ready to use with PLINK. These corrections are performed with the correct-files module. If users are combining multiple datasets, they can skip this module, as the combine-datasets module presumes this step has not been taken and performs this step.

## Combine-datasets module

**Fig. 10:**
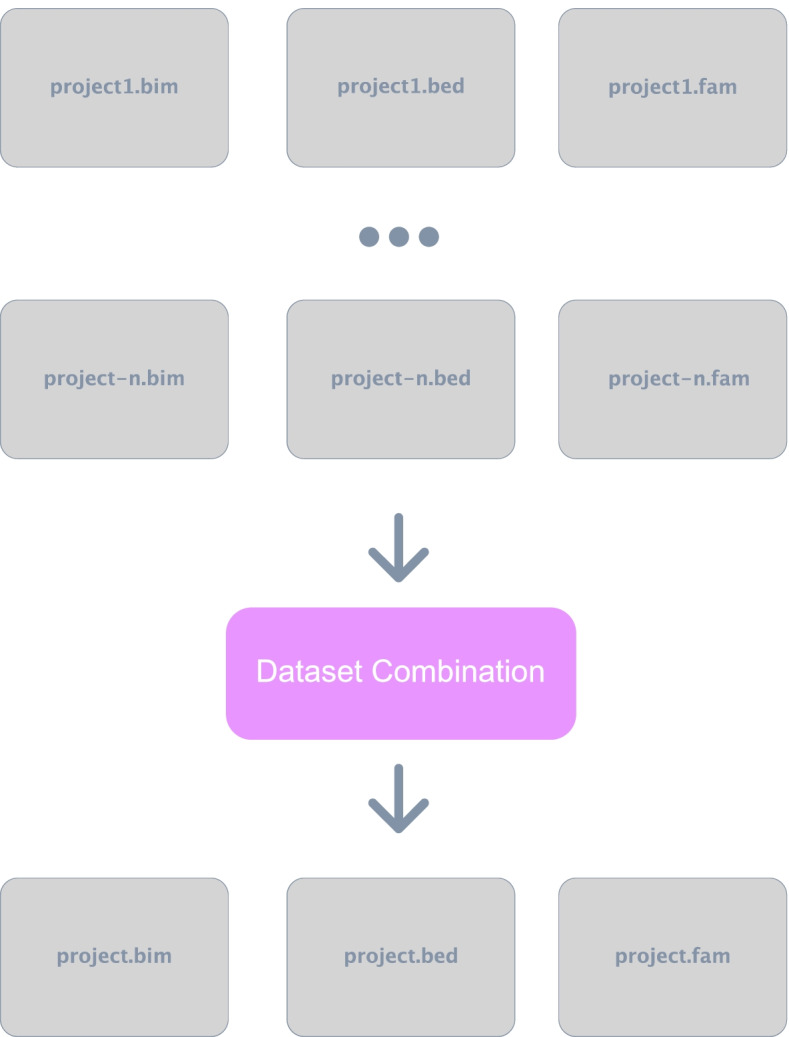
Combination of multiple datasets. Using the PLINK1 version files, *Canary* generates a list of SNPs common to all input datasets, it then uses PLINK1 merge datasets function to combine the datasets into a new dataset

The combine-datasets module of *Canary* combines multiple studies into a single dataset based on SNPs common to all datasets. It is important to note that this function simply combines data that are already harmonized, such as the sub-studies of the WHI data, and it does not perform the harmonization. Therefore, users’ discretion is advised. In this module, the output from the first module should be used as the input. First, this module generates a list of SNPs contained in each dataset, and then it compares each list to generate a list of SNPs common to all datasets. It then uses PLINK to merge the datasets and include only the SNPs common to all of the datasets. In a similar manner to how the convert-module completes multiple steps with a single line of Bash code, this combine-datasets module takes dataset directories, desired project name, and output directory as input and performs the steps in Fig. 10 automatically. It is important to note that this step is currently only available for PLINK1 file types, not PLINK2, as PLINK2 has not yet implemented this feature.

## Correct PLINK files

Once the dataset has been prepared, using only the convert-mac-module or the convert-mac-module followed by the combine-datasets module, the PLINK files must then be corrected to include the correct sex information about the individuals. To achieve this, the user must first follow the guide to create the phenotype file. Once they have done so, the user can use the update-sex function with PLINK to update this information in the dataset. For detailed instructions on how to perform this step, we advise users to check the PLINK documentation.

## Performing GWAS

After following the above steps using *Canary,* the resulting files are now ready to be used in a GWAS analysis. Performing sophisticated GWAS and other analyses automatically are currently out of the scope of this tool. However, the *Canary* container has PLINK and other GWAS related tools installed inside so that the user can perform analyses, particularly on an HPC, more easily. Additionally, others have previously published an excellent guide for performing quality control and GWAS analysis with PLINK version 1 [23]. *Canary* automatically downloads the code from this tutorial so that novice users can follow this guide. Moreover, although we advise users to follow the guide manually, we also provide a module, PLINK-QC, which will perform the QC steps outlined in this repo automatically. A full list of the installed software and packages are available at the GitHub repo at https://github.com/anb94/Canary.

## Conclusion

In conclusion, *Canary* can automatically convert MaCH imputed dosage files into PLINK-compatible files. Thus, providing an easier user-experience with MaCH dosage files, particularly novice users. Moreover, it is a singularity container that has multiple software pre-installed that are needed for conducting GWAS studies, thereby further reducing the number of hurdles for novice users to perform GWAS on HPC environments.

## Availability and requirements

Project name: Canary.

Project home page: https://github.com/anb94/Canary

Operating system(s): Platform independent.

Programming language: Shell & Python3.

Other requirements: SingularityCE v3.9 or newer.

License: MIT License.

Any restrictions to use by non-academics: None.

## Data Availability

*Canary* along with detailed instructions can be found at https://github.com/anb94/Canary. This tool is a singularity container and therefore requires singularity installed locally. The conversion tool is written in Shell. The dataset used as an example herein is the Women's Health Initiative Clinical Trial and Observational Study. The study data can be downloaded from the dbGaP web site, under phs000200.v12.p3 (https://www.ncbi.nlm.nih.gov/projects/gap/cgi-bin/study.cgi?study_id=phs000200.v12.p3), if granted approval. The most recent version of Canary can be found on GitHub at https://github.com/anb94/Canary. The archived version referenced in the manuscript can be found on GitHub at https://github.com/anb94/Canary/tree/canary_archive.

## Abbreviations

dbGaP: Database of genotypes and phenotypes
GARNET: Genomics and Randomized Trials Network
GUI: Graphical user interface
GWAS: Genome-Wide Association Studies
HPC: High-performance computing
NIH: National Institute of Health
SNP: Single-nucleotide polymorphism
SNV: Single-nucleotide variant
SHARE: SNP Health Association Resource
UKB: UK Biobank
WHI: Women’s health initiative
WHIMS: Women’s health initiative memory study
WGS: Whole-genome sequencing
